# Navigating the ethical “space in-between” nurses’ lived experiences in forensic inpatient care interpreted through Løgstrup’s ethical philosophy

**DOI:** 10.1080/17482631.2025.2514520

**Published:** 2025-06-05

**Authors:** Lars Hammarström, Siri Andreassen Devik

**Affiliations:** a Department of Health Sciences, Mid Sweden University, Sundsvall, Sweden; b Faculty of Nursing and Health Sciences, Nord University, Namsos, Norway

**Keywords:** Encounters, forensic nursing, lived experience, Løgstrup, nursing, reflective lifeworld research

## Abstract

**Purpose:**

This study examines the nurse—patient relationship in forensic psychiatric care (FPC) from a philosophical perspective, with a focus on ethical complexities. Using Løgstrup’s ethical philosophy, the work explores how trust, moral responsibility, and relational tensions shape caregiving.

**Materials and methods:**

A theoretical analysis was conducted based on five empirical qualitative studies of nurses’ lived experiences in forensic inpatient care. These studies, rooted in phenomenology and hermeneutics, were re-analysed using reflective lifeworld research (RLR), a phenomenological approach grounded in the lifeworld theory of Husserl and Merleau-Ponty, that emphasizes openness and reflection to capture the meaning of lived experiences. The analysis was interpreted through Løgstrup’s ethical framework.

**Findings:**

Five key themes emerged: Having Trust or Feeling Distrust, Being Compassionate or Being Indifferent, Having Courage or Being Afraid, Being Genuine or Pretending, and Being a Ballerina or Being a Bulldozer. These themes highlight the “space in-between”, where nurses navigate ethical tensions, institutional constraints, and patient interactions.

**Conclusions:**

Forensic psychiatric nursing requires balancing institutional control and compassionate care. Ethical encounters emerge through both self-reflection and relational engagement. Structured reflection and dialogue help nurses navigate ethical challenges, foster professional growth, and enhance patient-centred care.

## Introduction

This article offers a secondary analysis of previous studies and examines the nurse—patient relationship in forensic psychiatric care (FPC), presenting a philosophical reflection on caregiving and treatment in this setting. Taking the lifeworld perspective as its foundation, the discussion highlights how nurses’ encounters with patients are deeply intertwined with self-reflection. Because FPC involves substantial intervention in a patient’s autonomy and daily existence, it raises ethical considerations that require ongoing critical evaluation (Vincze et al., 2015).

Knud Ejler Løgstrup (1905–1981), a Danish professor of ethics and religion, explored human interdependence through a phenomenological lens, and he emphasized the unspoken ethical obligation that emerges when people encounter each other (Løgstrup, 1997). Løgstrup’s philosophy stresses that human encounters, by nature, are not problems to be solved but interactions that demand reflection (Martinsen, 2012). His concept of the ethical demand is underpinned by the fact that human beings exist within a shared reality, where they rely on one another for personal and collective development. This demand is silent yet radical: it does not dictate specific actions, but rather calls for a deep sense of responsibility in relation to the other. Løgstrup (1997) suggests that to truly engage in an ethical encounter, one must be willing to act in the best interest of the other.

Nursing in FPC entails inherent ethical complexity, as it involves caring for individuals who have lost their personal freedom. Nurses must continuously navigate a balance between caregiving and matters of security, managing the unevenness that characterizes their relationship with patients (Jacob, 2012). The duality of their role demands that they prioritize patient well-being while also ensuring the safety of the patient, staff, and society (Hörberg, 2008). This unique context presents significant challenges, as nurses engage with patients who may have violent histories, severe mental health conditions, and complex legal constraints (Laiho et al., 2016). Despite the security-focused environment, therapeutic relationships must still be built on trust, which is a fundamental component of caregiving (Gildberg et al., 2012).

Løgstrup (1983b) outlines how individuals instinctively resist intrusion, violation, or exposure, which he conceptualizes as untouchable zones. Without sensitivity to these personal boundaries, meaningful encounters become difficult. A nurse must develop an awareness of what they share with the patient—i.e., recognize the human connection despite differences—in order to foster openness and trust (Devik et al., 2013). This requires an openness to the other’s experience, where preconceived notions are set aside and the nurse allows the patient’s expressions to make an impression (Barker & Buchanan-Barker, 2005; Brookes, 2005). Løgstrup (1983b) further argues that these expressions cannot be controlled but only perceived, which requires an ethical attentiveness that enables genuine relational engagement.

While legal frameworks and official policies govern FPC, certain aspects of human existence remain beyond human construction, including trust, mercy, openness, hope, joy, and love; Løgstrup (1997) describes these as “sovereign utterances of life”. A caregiving approach that embraces these inherent human qualities enables deeper understanding and patient-centred care, as it allows the nurse to see beyond the patient’s actions and recognize their humanity. Løgstrup (1997) advises against disregarding these essential conditions, arguing that human beings must actively reflect on and regulate their impulses in order to truly perceive and respond to the other.

According to Løgstrup (1997), trust is the foundation of human interaction, and he suggests that individuals initially approach one another with openness—unless given a reason to withdraw. In FPC, where hierarchical structures are deeply embedded, trust is particularly fragile. If trust is eroded, coercion and control may replace genuine caregiving, negatively impacting both the nurse—patient dynamic and the overall therapeutic environment (Berry & Robertson, 2019).

A key concept in phenomenology is the tension between opposites, which characterizes the way humans experience the world. Husserl (1970) describes the lifeworld as shaped by interactions between contrasting forces, such as trust and distrust, closeness and distance, authority and vulnerability. This interplay is particularly evident in FPC, where nurses must constantly recalibrate their ethical stance and strive for a balance between emotional involvement and professional detachment. H. Dahlberg (2019) refers to this dynamic as “the space in-between”—i.e., the fluid movement between opposing yet interconnected states that defines human interactions.

In this context, a nurse may simultaneously experience trust and distrust towards a patient: the trust may emerge from compassion and professional confidence, while the distrust may be rooted in unpredictable behaviours or prior experiences (Hammarström et al., 2020). Similarly, compassion and emotional distance coexist, as nurses remain empathetic while also protecting their own well-being in a high-risk environment. These contrasts are not contradictions but rather relational dynamics that are central to the complexity of forensic psychiatric nursing (H. Dahlberg & Dahlberg, 2019).

To this end, this article aims to examine nurse—patient encounters in FPC using philosophical perspectives and empirical findings (Hammarström et al., 2019, 2020; 2023; Hammarström, Devik, Häggström, et al., 2022; Hammarström, Devik, Hellzén, et al., 2022). Drawing on previous research within the Reflective Lifeworld Research tradition, particularly the theoretical foundation laid by Hörberg and Dahlberg (2015) and Løgstrup’s ethical philosophy as an analytical framework, the article will explore how trust, moral responsibility, and relational tensions influence caregiving in this specialized setting. By applying these philosophical perspectives, the study seeks to enhance the understanding of the ethical challenges that define forensic psychiatric nursing.

## Materials and methods

In line with the structure of Dahlberg and Hörberg (2015), the empirical findings from previous studies were analysed through a theoretical lens. The five studies (Table I) forming the empirical foundation are all rooted in the concept of the lifeworld and employ methods grounded in ontological perspectives within hermeneutics and phenomenology. The overall aim of the analysed empirical qualitative studies was to gain a deeper understanding of nurses’ lived experiences of encounters with patients with mental illness in forensic inpatient care.

**Table I.** Overview of the five published studies that constitutes the empirical foundation.

Paper	Aim	Participants	Data collection	Analysis method
Hammarström et al. (2019)	Illuminating the meaning of nurses’ lived experiences of encounters with patients with mental illnesses in forensic inpatient care.	2 RNs^1^, 3 SRNs^2^ and 8 ASNs^3^.	Narrative interviews	Phenomenological-hermeneutic approach (Lindseth & Norberg, 2004).
Hammarström et al. (2020)	Explore and interpret nurses’ experiences of compassion when caring for patients with mental illness in forensic psychiatric inpatient care.	2 RNs^1^, 3 SRNs^2^ and 8 ASNs^3^.	Secondary supplementary analysis of paper I, 2019, narrative interviews	Hermeneutic approach (Fleming et al., 2003).
Hammarström, Devik, Häggström, et al. (2022)	To illuminate the essential meanings of carers’ lived experience of regulating oneself when caring for patients with mental illnesses in forensic inpatient care.	1 RNs^1^, 5 SRNs^2^ and 3 ASNs^3^.	Narrative interviews	Phenomenological-hermeneutic approach (Lindseth & Norberg, 2004).
Hammarström, Devik, Hellzén, et al. (2022)	To describe the phenomenon of vulnerability as experienced by carers in forensic inpatient clinics.	3 RNs^1^, 3 SRNs^2^ and 3 ASNs^3^.	Narrative interviews	Reflective lifeworld research (K. Dahlberg et al., 2008).
Hammarström et al. (2023)	To describe the phenomenon of “fleeing the situation” in patient encounters as described by carers in forensic inpatient care.	3 RNs^1^, 3 SRNs^2^ and 3 ASNs^3^.	Narrative interviews	Reflective lifeworld research (K. Dahlberg et al., 2008).

### Participants and data collection

The five studies were all carried out in a major forensic clinic in Sweden. The clinic had approximately 180 employees and 100 patients across eight wards—each housing approximately 12 to 15 patients—at the time this study was conducted. Most patients were men aged 25–45 years who were convicted of some type of violent crime. Approximately 60% of the patients in this setting had schizophrenia or another psychotic disorder and had been transmitted to involuntary inpatient FPC due to some sort of violent crime rather than being incarcerated in the penal system, in accordance with the Forensic Mental Care Act (LRV 1991:1129).

Participants across the studies were all recruited from the same forensic clinic with the assistance of the first-line manager, through purposive sampling that set experience in FPC as an inclusion criterion. Written and verbal informed consent was obtained from the participants. In Hammarström et al. (2019, 2020), the sample included ten men and three women aged 28–67 years (Md = 36), with FPC experience ranging from five to 46 years (Md = 11); the group comprised five registered nurses (three of whom were specialists in psychiatric nursing) and eight assistant nurses with psychiatric training. Hammarström, Devik, Häggström, et al. (2022) involved nine participants (five men, four women), aged 30–66 years (Md = 41.6), with FPC experience between two and 32 years (Md = 12); this sample included five specialist nurses, one registered nurse, and three assistant nurses. Hammarström, Devik, Hellzén, et al. (2022) also had nine participants (four men, five women), aged 31–67 years (Md = 39), with FPC experience spanning three to 33 years (Md = 13). In Hammarström et al. (2023), the nine participants consisted of four women and five men, and whose ages ranged from 30 to 66 years (Md = 41.6); their experience in forensic inpatient care ranged from 2 to 32 years (Md = 12). In total, the empirical data consisted of interviews with 40 participants.

In the studies, narrative individual interviews were conducted with forensic mental health care staff to explore their experiences and emotions concerning patient encounters. The interviews consisted of open-ended questions and were conducted either in person or digitally, depending on pandemic restrictions. All interviews were transcribed verbatim, with non-verbal cues included so as to capture the full depth of the participants’ experiences.

### Ethical considerations

This study was conducted according to national law with the approval of the Swedish Ethical Review Authority (No. 2018/157–31) and follows the ethical guidelines regarding information, consent, confidentiality, and data use outlined by the Swedish Research Council (2017). The study adheres to the principles of the Declaration of Helsinki (WMA. World Medical Association, 2013).

The five original studies have all been published and were conducted and authored by the same research team, ensuring continuity in methodology and epistemological grounding. As the study draws on previously collected material, the ethical considerations were primarily focused on handling and analysing the data with respect for participants’ anonymity and integrity. All data was securely stored, and measures were taken to ensure confidentiality. Participation in the original data collection was voluntary, with informed consent obtained written and verbally prior to data collection.

Given the study’s phenomenological foundation, emphasis was placed on openness and ethical sensitivity throughout the analytical process. Respect for the participants’ lived experiences remained central, which ensured that their voices were represented accurately and responsibly.

### Results from the empirical studies

In forensic psychiatry, achieving core values in patient treatment requires seeing the person behind the crime. This involves meeting patients with authenticity, compassion, and trust, as well as having the courage to remain present in the situation with a caring approach that prioritizes the patient’s best interests.

At the same time, encounters with patients are characterized by the duality of acting on the patients’ needs while managing the rules and norms stipulated in laws and regulations governing FPC and societal protection. Encounters in FPC and being a nurse are intertwined with being put in a position of power, being fragile, dealing with fear and disgust, and not having to adopt a role that does not align with one’s true self.

An encounter entails being in a duality and having the insight of the “space in-between” these feelings and the dual mission that underpins caring in FPC. In the space in-between, there is room and potential for the nurse’s personal growth, to achieve a phenomenological attitude and to truly embrace the patient’s lifeworld.

### Forming a general structure

The analysis was based on published findings from the original studies. No re-transcription or re-coding of raw data was performed; instead, key meanings and structures were thematically summarized to support the theoretical elaboration. The findings from the five empirical studies prompted further inquiry into the shaping of encounters in FPC. To deepen our understanding, we re-examined these findings, analysing their essential meanings collectively through a new phenomenological meaning analysis. This approach sought to establish a general structure through following the methodological principles of reflective lifeworld research (RLR) (K. Dahlberg et al., 2008). This type of analysis serves as an integrative synthesis that draws together and abstracts the initial findings so as to unify the different nurses’ perspectives. The focus of this analysis was the phenomenon of “encounters in a forensic psychiatric setting.” Additionally, a philosophical examination was conducted to deepen understanding of this phenomenon. According to K. Dahlberg et al. (2008), theoretical or philosophical materials should not be part of the initial empirical analysis in phenomenological studies, as strong theories might overshadow the subtle nuances of participants’ experiences; instead, RLR recommends using theories and philosophy to further explore the findings only after the empirical analysis has been conducted. This approach strengthens the lifeworld data, as it allows for more profound and creative insight into the phenomenon.

### A new analysis

The analysis to develop a general structure began with an open reading of the essential meanings derived from the five empirical studies, guided by such questions as “What characterizes encounters in forensic psychiatric care?” and “How can these encounters be understood as a practice of caring?” In line with the principles of RLR, the analysis entailed movement between the whole and its parts, conceptualized as “figure and background” (K. Dahlberg et al., 2008). Specifically, patterns of meaning from each study were examined against the others, with each study’s findings serving as a figure that highlighted aspects of the others. This interplay of patterns was explored in varying combinations to uncover new, abstract structures of meaning. The analysis process maintained an openness and sensitivity to the phenomenon under study and to the original lifeworld experiences, with the researchers adopting a “bridling” attitude to deepen their understanding (K. Dahlberg et al., 2008). Ultimately, a general structure emerged that shed light on the essential meanings of encounters in FPC based on the lived experiences of nurses. The subsequent stage extended this analysis by incorporating insights from Løgstrup’s ethical perspectives. In line with the RLR approach, Løgstrup’s ethical concepts were applied post hoc, during the reflective phase of analysis, to deepen the interpretation of the emerging essential meaning”.

## Findings

First, the general structure of the findings of the five empirical studies is described, followed by the philosophical examination, in which there are five components: Having Trust or Feeling Distrust, Being Compassionate or Being Indifferent, Having Courage or Being Afraid, Being Genuine or Pretending, and Being a Ballerina or Being a Bulldozer.

### General structure

#### The “space in-between” in FPC

Encounters with patients in FPC are not only characterized by the patient’s expression of suffering but also by the duality of caring for both the patient’s needs and societal protection (Hammarström et al., 2019). Caring for patients in forensic psychiatry means being faced with several situations that threaten the nurses’ professional identity. By letting the patient’s expression make an impression—taking a step back to be able to take a step forward by regulating their emotions (i.e., by using strategies that create temporary distance)—it is possible to get closer to patients to alleviate the suffering. Even though they sometimes face threats, violence, and humiliation, nurses make decisions grounded in compassion and the patient’s needs (Hammarström et al., 2020).

The “space in-between” refers to the shared experience that arises when two people meet, especially in a caring relationship. According to H. Dahlberg (2019), this space is shaped by both similarities and differences, by trust and uncertainty, and by the balance between emotional connection and professional distance. It is the space where the patient’s expressions leave an impression on the nurse and where the nurse, in turn, responds not just as a professional but as a fellow human being. Instead of seeing the dualities that constitutes the space in between as opposites, they should be understood as part of the same whole. By becoming aware of this space and embracing its tensions, both the patient and the nurse can experience a deeper sense of understanding and care (Holopainen et al., 2015).

#### Having trust or feeling distrust

Encountering patients in FPC involves developing trust and managing the fluctuating feelings of distrust that may arise. Nurses must remain vigilant for potential threats as well as foster an environment of predictability to enable patient participation and symmetrical relationships. Openness and avoiding prejudices are crucial in this approach, in line with extant research on setting aside emotions that hinder trust development (Løgstrup, 1997). Building a bond based on trust is a time-consuming process marked by numerous encounters that can evoke positive or negative emotions, which impact the development of trust and the caring relationship as illustrated by one of the participants in Hammarström et al. (2019).

For me, I think the key has been that I managed to create trust through my encounters. You should meet his needs and listen. He should feel involved.

Nurses recognize the need to be accountable to and supportive of their patients, which they view as essential to their caregiving role. Being trustworthy becomes a means through which to invite patients to share their suffering, with an emphasis on acceptance. This necessitates nurses’ introspection on their vulnerabilities and regulation of their emotions to avoid compromising trust. Maintaining openness and truly seeing and understanding the other in the encounter are crucial aspects (Hammarström et al., 2019).

At the same time, nurses grapple with reminders of patients’ criminal histories, which contribute to distress and nurture distrust (Hammarström et al., 2020). Nurses often rely on institutional rules and regulations for protection, which influence their perceptions of encounters and hinder trust development. The findings suggest that displacing trust to the relationship, rather than rules, may reduce aggression and coercive actions (Hammarström et al., 2020) as well as empower nurses to meet patients’ expectations of care and trustworthiness (Løgstrup, 1997).

#### Being compassionate or being indifferent

Understanding and responding to patient expressions is crucial to developing compassion and recognizing them as individuals (Hammarström et al., 2019). However, decoding these expressions—which range from verbal outbursts to apathy—can lead to nurses feeling frustrated and the risk of indifference (Hammarström, Devik, Häggström, et al., 2022).

In FPC, where rules often take precedence over patient participation, the findings indicate that nurses’ actions within this framework may still be rooted in genuine compassion, even when they are following rules (Hammarström et al., 2019). Balancing compassion and paternalism is especially complex given the dual nature of FPC (Hammarström et al., 2020), and it challenges the perceived divide between rule-based actions and compassionate responses (Løgstrup, 1987).

Although caring encounters are complex, they may still evoke feelings of discouragement, shame, and failure, which in turn can lead to indifference in the nurse—patient relationship (Hammarström, Devik, Häggström, et al., 2022). Reflecting on one’s own responsibilities can bring clarity to such challenges (Løgstrup, 1983a); additionally, promoting a permissive environment for nurses to address their own suffering may prevent self-blame and judgement (Hammarström, Devik, Häggström, et al., 2022). The findings emphasize a self-compassionate approach that supports nurses’ well-being and provides tools to understand patients’ expressions of suffering without conflating personal and patient experiences (Hammarström et al., 2022).

#### Having courage or being afraid

Caring for patients in FPC could entail facing fear, especially when patients’ expressions of suffering pose potential dangers (Hammarström et al., 2019). Nurses acknowledge patients’ needs but grapple with their own fear, leading to an internal struggle between staying with the patient and self-preservation (Hammarström et al., 2020). Fear complicates understanding encounters and challenges sustained commitment to the other’s well-being (Løgstrup, 1987).

Persisting in caring situations may inspire change, foster compassion, and thus possibly provide meaning to the patient’s life (Hammarström et al., 2019). This courageous approach requires the strength to be open and allow the other to get close (Løgstrup, 1997). Nurses link fear to uncertainty and to a lack of confidence in responding to ambiguous expressions of suffering, in both patients and themselves (Hammarström et al., 2020). Fear may hinder the normalization of patients, leading to a process of either inclusivity or exclusivity (Løgstrup, 1987). Negative patient representation stems from immediate danger and the crimes they committed (Hammarström et al., 2022): here, patients are perceived as potential risks, which prompts nurses to devise safety strategies (Hammarström et al., 2019).

Overcoming fear requires both confidence and reflective engagement with one’s own vulnerability. This process is strengthened through dialogue with fellow nurses, as shared experiences help in navigating the emotional and ethical challenges of care. In this context, Løgstrup’s (1997) understanding of trust as a fundamental condition in human encounters and Martinsen’s (2006) emphasis on the ethical responsibility of care are particularly relevant, as they highlight the relational and existential dimensions of overcoming fear (Hammarström et al., 2022).

Courage manifests in the capability and motivation to help others in need face vulnerability and suffering (Løgstrup, 1997). Nurses verbally express fear, which underscores the intertwining of courage and self-perceived vulnerability that is crucial for ethical caregiving (Hammarström et al., 2019). Courage means not just witnessing patients’ suffering but also trusting oneself, addressing one’s vulnerabilities, and growing as a person (Løgstrup, 1997).

#### Being genuine or pretending

Caring in FPC is intricate, as it entails a delicate balance between ensuring safety and embracing a therapeutic, patient-centred approach (Hammarström et al., 2023). Encounters in FPC often involve managing the contradictory roles of caring and guarding, where guarding in form of control dominates interactions with patients, situations, fellow nurses, and oneself, as illustrated by a quote from one of the participants from Hammarström et al (2022).

It was kind of weird when you walked around and played tough. Over time you felt more secure with the job, with the patients and also with your co-workers, it felt more right and was usually better if you let your shoulders down a little and was more natural … I also notice that my job becomes easier, and it goes better with the patients when they notice that I am genuine … we connect in a better way.

Nurses navigate diverse encounters, which underscores the importance of controlled responses that project calm and comfort (Hammarström et al., 2022). Coping with contradictory emotions, nurses often adopt a protective façade, one linked to their dependence on others and the ever-changing dynamics in the ward (Hammarström et al., 2019). Approaching patients involves a self-perception rooted in trust and confidence of acceptance despite one’s shortcomings (Hammarström et al., 2022). The findings underscore the significance of time and experience in finding one’s place, understanding oneself, and acting authentically in encounters. Nurses recognize the importance of belonging to a community for trust and safety, even if it means adapting to a new culture and adopting a role that they perceive is expected of them (Hammarström et al., 2020).

Disguising vulnerability is connected to conforming to FPC regulations at the expense of authenticity (Hammarström et al., 2019). Control is a pivotal aspect of nursing in FPC, where nurses must intervene and engage with potentially threatening individuals—which necessitates internal emotion control and a professional façade (Hammarström et al., 2022).

However, the findings also reveal that having an open mind and embracing others’ otherness aids nurses in building trust, forming alliances, and fostering authentic connections with patients (Hammarström et al., 2019; Hammarström, Devik, Hellzén, et al., 2022). This openness allows for a stronger bond, where nurses navigate the space between being genuine and pretending as well as address both their own and the patient’s vulnerabilities (Hammarström et al., 2022). Contrary to the potential risks, this indicates that being authentic and genuine with patients is crucial, as it fosters trust and acceptance (Løgstrup, 1997).

### Being a ballerina or being a bulldozer

In encounters between nurses and patients, nurses often use specific approaches, which are described in metaphors that stem from the narratives of the participants in Hammarström et al. (2023). One such metaphor is the “bulldozer approach,” wherein the nurse adopts a protective stance, acting as a shield to ensure what they perceive to be a safe and secure environment for both patients and staff. Conversely, the metaphor of the “ballerina approach” represents a way of fostering a sense of security, trust, and closeness by engaging with the patient, setting aside time, and demonstrating openness and accessibility.

These metaphors—frequently mentioned in the interviews with nurses—highlight the challenges and forms of resistance that nurses experience in their encounters. Nurses report feeling fear in response to threats, facing crucial decisions alone, attempting to maintain control over their circumstances, and navigating the inner conflict between remaining present versus withdrawing from a challenging situation (Hammarström et al., 2023).

This concept of retreating or “fleeing” from an encounter when confronted with resistance is revealed to be a complex phenomenon that is deeply tied to nurses’ sense of self as professionals. Løgstrup (1983a) states that, to fully grasp and make sense of what moves us, we must consciously reflect on our responses—a process that often requires gaining some distance in order to facilitate understanding.

Nurses often find that what starts as a typical encounter may unexpectedly transform into a threatening or hazardous situation, which presents a dilemma: they recognize the encounter’s potential for contributing positively to long-term relationships, yet it is sometimes perceived as intolerable (Hammarström et al., 2023).

In clinical settings, difficult patient interactions can impact a nurse’s self-concept, sometimes leading to feelings of shame and self-blame. Consequently, the shift in an encounter’s tone is not merely a question of endurance: it connects to the nurse’s professional self-perception, their patience, and concern about maintaining control in their professional role (Hammarström et al., 2023). In this context, “fleeing the encounter” can be seen as a means of reconnecting with oneself, navigating between presence and withdrawal, and finding equilibrium amid the tension of chaos and stability. This balance entails both closeness and distance, being together yet retaining solitude (Løgstrup, 1983a).

### Synthesis of the meaning of “the space in between”

**Figure 1.**
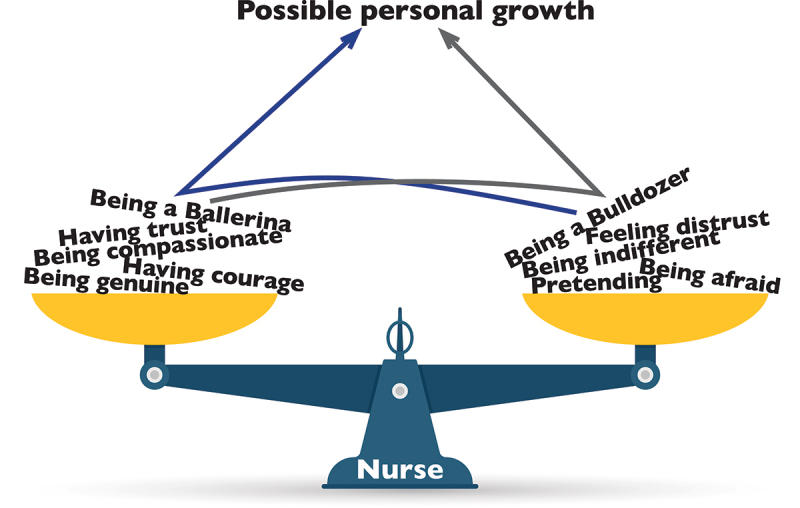
The encounter and the space in-between.

Together, the five themes describe how the space in between takes shape as a relational and ethical dimension in FPC. This space is not defined by fixed positions such as trust or distrust, compassion or indifference, or control versus closeness. Rather, it emerges in the ongoing movements between these positions. Nurses continuously adjust their presence in relation to patients, navigating emotional, moral, and ethical demands. What unites the themes is a shared sense of tension and responsiveness, as nurses respond to suffering while also protecting themselves, they seek authenticity while adapting to institutional expectations. The space in between becomes visible in these shifting movements, where care is not the absence of conflict, but the ability to remain present within it—allowing the patients’ expressions to make an impression. In doing so, within a setting shaped by risk, coercion, rules, and asymmetry, nurses create conditions for mutual understanding with a possibility for personal growth which is illustrated by the figure (Figure 1) below.

## Discussion

The theoretical perspective that emerged in the analysis is based on the independent and distance-free access to the world and the context in which one is embedded (*Danish: Sansing)* (Løgstrup, 1997). This article shows that encounters with forensic psychiatric patients can and should be seen as being permeated by communication, where the nurse is the key person in the creation of a caring relationship through an attempt to share the patient’s lifeworld (Martinsen, 2012). The findings illustrate how in encounters, nurses must manage the duality that exists in FPC and the “space in-between” their lifeworld that occurs in the impressions of the encounter. A patient‘s expression affects the nurse‘s impression, which helps us grasp not only what the nurse is affected by in the encounter but also what is expressed in the awareness of the impression given by the encounter. This provides an understanding of the meaning of the encounter through the eyes of the person behind the keychain. This article elucidates how an encounter contains several existential phenomena that belong to the basic conditions of life.

Furthermore, it appears that a nurse’s experience of an encounter is affected by the tone of the impression. Heidegger (1927/2010) explains tone as something that opens the world and helps one to be in harmony with it. Heidegger’s definition implies that, in an encounter, it is impossible to distinguish between the person’s consciousness and the world they are surrounded by, as the tone originates in the social and natural environment and not in something the person has created themselves. To this end, nurses are structurally and contextually affected not only by the duality of FPC but also by the culture that exists at the clinic and the ward.

The impression of the encounter seems to be crucial to the experience of, e.g., vulnerability, as one’s relationship with others can increase or decrease the feeling of being exposed. Therefore, the encounter can also be seen as a relational phenomenon. An encounter with a patient means that the nurse is affected by something that concerns they which can be seen as an acknowledgement of the content of the impression given by the patient’s expression (Løgstrup, 1983a). The patient’s expression is sometimes met with resistance, as there is always an initial desire to reshape the impression and intervene based on one’s own assumptions (Løgstrup, 1983b). To understand the impression, distance is required—a distance that is not about getting out of the encounter and looking at it from the outside, but rather creating distance from one’s naturalistic intention. If nurses are emotionally present, sensing opens them up to understanding the impression (Løgstrup, 1983a).

Løgstrup (1983b) asserts that all living beings resist being invaded, violated, or having what he refers to as their “untouchable zones” exposed. Without openness and insight concerning these untouchable zones, they become barriers that hinder genuine encounters. It is only when the nurse—through understanding and interpretation—senses what is shared by the patient, and recognizes oneself despite differences, that the nurse can lower their guard, reveal their true self, and enter a room of awareness (Devik et al., 2013). Encountering others with the intention of allowing them to raise their voice and tell their story requires openness—i.e., being receptive to the impressions they leave—instead of acting solely upon one’s own perceptions and experiences (Barker & Buchanan-Barker, 2005). Løgstrup (1983b) shares this reasoning in his discussion on the phenomena that arise in the impression of expressions: since these phenomena can only be understood through openness and presence, they provide insight into the fact that impressions are something we can follow but never fully control.

As Løgstrup (1997) argues, trust is one of the fundamental conditions of human life, as it shapes our ethical responsibilities towards one another. We continually place parts of our lives in the hands of others, relying on them to handle this gift with care and responsibility. At the same time, ethical encounters also require trust in oneself, wherein self-reflection and authenticity are crucial to acting with integrity (Løgstrup, 1983a). Additionally, our relationships are shaped by trust in the surrounding environment, namely the belief that it supports and enables ethical actions. When trust is nurtured, it fosters mutual understanding and responsibility, but when it is broken, it affects both an individual’s self-perception and their relationships with others (Løgstrup, 1997). Hence these findings may inform both nursing education and organizational ethics by highlighting the need for reflective training and support structures that help nurses navigate the ethical complexity of encounters (Hammarström et al., 2019, 2020; 2023; Hammarström, Devik, Häggström, et al., 2022; Hammarström, Devik, Hellzén, et al., 2022).

However, trust alone is not always sufficient: ethical responsibility also demands courage. As Thorup et al. (2012) argue, courage is an essential quality in care ethics, as it enables individuals to remain present in moments of suffering and vulnerability. The authors describe how acting with care often requires the courage to face uncertainty, to step into emotionally demanding situations, and to confront one’s own limitations. Without courage, the weight of ethical responsibility may lead to avoidance rather than engagement, ultimately weakening the trust that forms the foundation of human relationships. In this sense, courage does not oppose trust but rather reinforces it, enabling ethical actions to be carried out with integrity even in the face of difficulty (Thorup et al., 2012).

In FPC, where control and structure often dominate, this trust is constantly challenged. Legislation shapes the care by incorporating both human-made and intrinsic constructs that influence every individual. However, as Martinsen (2012) highlights, not all aspects of care are socially constructed: some elements—such as vulnerability, interdependence, and the finite nature of life—are pre-cultural conditions that are essential to human existence. Løgstrup (1997) emphasizes these sovereign utterances of life (trust, mercy, hope, joy, and love), which, although inherent, can be either nurtured or suppressed in human interactions (Martinsen, 2006). When the space is restricted, conditions such as hopelessness and alienation can emerge; this illustrates how structural and institutional settings directly impact these fundamental life expressions.

Power and powerlessness are ethical issues that nurses must address (Hammarström et al., 2023). As Kristensson Uggla (2022) highlights, power is always present in the patient—caregiver relationship, with the patient placed at a disadvantage institutionally, existentially, and cognitively. Like the patient—caregiver relationship itself, power is dynamic. Holding this power and responsibility for another’s suffering can be likened to having a “proxy” (Rundqvist, 2004). Løgstrup (1997) argues that human encounters inherently involve trust and vulnerability, connoting that the one who holds power—here, the nurse—must acknowledge the ethical demand that arises in the relationship. Trust is initially given but can be lost through misuse; thus, power in a caring relationship requires a responsible approach, wherein the nurse must protect the fragile trust that underpins the relationship. A patient’s resistance should not be seen as opposition to authority but as an attempt to maintain dignity and agency. Accordingly, the nurse’s role is not to suppress this but to respond in a way that fosters understanding and care (Skjervheim, 1996).

Based on the empirical foundation of Hammarström et al. (2019, 2020; 2023; Hammarström, Devik, Häggström, et al., 2022; Hammarström, Devik, Hellzén, et al., 2022), talking about their emotions allows nurses to reflect on their own experiences, which in turn encourages patients to participate more actively in the caring relationship. This openness can empower patients and support their engagement in care. For nurses, being given the opportunity to verbalize emotionally challenging encounters and personal shortcomings can create a path towards personal growth and a deeper understanding of the patient’s perspective. Structured reflection plays a key role in this process. It enables nurses to recognize and address their own vulnerabilities, helping them regulate emotional responses in complex or demanding situations. This reflective work is particularly important when navigating the space in-between, where ethical tensions, emotional strain, and professional responsibilities meet. By challenging their own assumptions and deepening their understanding of their role as nurses, they may enhance their well-being and strengthen their capacity to provide mental health care grounded in authenticity, responsiveness, and trust (Hammarström et al., 2019, 2020; 2023; Hammarström, Devik, Häggström, et al., 2022; Hammarström, Devik, Hellzén, et al., 2022). This understanding aligns with Patel and Metersky’s (2022) concept analysis, which underscores how structured reflective practice helps nurses recognize and process emotional, ethical, and professional tensions in real-time. Their findings highlight the importance of reflection-in-action and reflection-for-action as dynamic strategies to navigate the “space in-between”, fostering both patient empowerment and professional growth.

In **conclusion**, the duality of FPC and the “space in-between” that arises in the impressions of encounters mean that the nurse is confronted with existential phenomena that constitutes one’s lifeworld. These impressions of encounters entail facing one’s own fragility and what is perceived as personal or private. Vulnerability can be a burden if it is not addressed and instead protected at all costs, or it can be a possibility for change through being authentic and true to oneself (Løgstrup, 1997). For nurses, this has meaning, as it highlights the dilemma of navigating between the rules and norms that stipulate FPC and the lifeworld of the patient, other nurses, and themselves. By being active in the “space in-between” and reflecting on openness, the nurse moves within this duality that exists in the continuum between opposite phenomena. The space in between contradictory impressions may become a place where the nurse may unravel their own lifeworld, as well as a place where one may grow as a person. According to Løgstrup (1997), openness and awareness of impressions, cultivated through active self-reflection, are essential for conveying meaning. Nevertheless, in line with his ethical philosophy, such reflection is not merely an internal process but is inherently relational; understanding the patient’s world and oneself is shaped in encounters with others, where verbalized and shared reflections play a crucial role. This establishes the need for dialogue and structured reflection in professional settings, to fully grasp the ethical demands present in human interactions (Løgstrup, 1997).

Although legal and organizational frameworks differ between countries, the ethical and relational core of patient encounters is shared across contexts. The findings of this article highlight the need for structured reflective practices within FPC, such as ethical case discussions and team-based dialogues, to support nurses in navigating the relational and ethical complexity of patient encounters. If the nurse can encounter the other through openness and compliance, it is possible that the meeting can become a place that promotes personal growth for the nurse, that provides space, and that encourages the sovereign utterances of life. In turn, this enriches nursing, because nurses can to a greater extent understand both themselves and patients’ expressions of suffering. This enables person-centred care based on the patient’s lifeworld, as it should be (Hammarström et al., 2022).

## Data Availability

Any underlying data will be shared upon reasonable request.
